# Aripiprazole Augmentation to Mood Stabilizers for Obsessive-Compulsive Symptoms in Bipolar Disorder

**DOI:** 10.3390/medicina57010009

**Published:** 2020-12-24

**Authors:** Gabriele Di Salvo, Giuseppe Maina, Enrico Pessina, Elena Teobaldi, Francesca Barbaro, Azzurra Martini, Umberto Albert, Gianluca Rosso

**Affiliations:** 1 Department of Neurosciences “Rita Levi Montalcini”, University of Turin, 10126 Turin, Italy; gabriele.disalvo@unito.it (G.D.S.); elena.teobaldi@unito.it (E.T.); gianluca.rosso@unito.it (G.R.); 2 Psychiatric Unit, San Luigi Gonzaga University Hospital, 10126 Turin, Italy; 3 Mental Health Department of Alba and Bra, 12042 Cuneo, Italy; enricopessina@hotmail.com (E.P.); barbaro.fra@gmail.com (F.B.); azzurramartini@libero.it (A.M.); 4 Psychiatric Section, Department of Medicine, Surgery and Health Sciences, University of Trieste, 34149 Trieste, Italy; ualbert@units.it

**Keywords:** aripiprazole, bipolar disorder, OCD, YBOCS, efficacy, adverse events

## Abstract

*Background and Objectives*: Aripiprazole is a first-line agent in the treatment of bipolar disorder (BD) and available data demonstrates its efficacy on clinical symptoms in serotonin reuptake inhibitors-resistant obsessive-compulsive disorder (OCD) patients. Therefore, aripiprazole augmentation to mood stabilizers could represent a promising treatment in BD patients with comorbid OCD. The study examined the efficacy and safety of aripiprazole added to lithium or valproate for the treatment of obsessive-compulsive (OC) symptoms in euthymic BD patients with comorbid OCD. *Materials and Methods*: This is a 12-week prospective observational study. The efficacy of aripiprazole on OC symptoms was assessed through the mean change of Yale–Brown Obsessive-Compulsive (YBOCS) total score. Tolerability was assessed with the Utvalg for Kliniske Undersogelser (UKU) side effect scale and by reporting adverse events. *Results*: A total of 70 patients were included in the analyses. The withdrawal rate was 21.4%, mainly due to adverse events. Mean ± SD final aripiprazole dose was 15.2 ± 5.3 in the completer sample (*N* = 55). The Y-BOCS mean score decreased from 24.0 ± 4.1 at baseline to 17.1 ± 4.3 at 12 weeks. Treatment response rate (Y-BOCS reduction ≥ 35%) was 41.8%, while partial response rate (Y-BOCS reduction greater than 25% but less than 35% from baseline) accounted for the other 18.2% of patients. Overall, 91.4% of completers had at least 1 adverse effect (tremor, tension/inner unrest, reduced duration of sleep, akathisia). No significant differences emerged comparing aripiprazole efficacy and tolerability between patients treated with lithium or valproate. *Conclusion*: Our findings show that aripiprazole addition to lithium or valproate can reduce OC symptoms in real-world BD euthymic patients.

## 1. Introduction

Obsessive-compulsive disorder (OCD) is one of the most common psychiatric comorbidities in bipolar disorder (BD). In population-based studies, the prevalence of OCD in BD patients was 11–21% [1,2,3]. OCD comorbidity impacts the clinical presentation and course of BD, as it is associated with earlier age at onset, increased prevalence of suicide attempts, psychotic symptoms, rapid cycling, alcohol dependence, and greater functional disability [4,5,6,7,8,9]. Moreover, BD with co-occurring OCD has been found to be associated with polypharmacy and poor treatment response [10].

The treatment of BD-OCD patients is particularly challenging: the serotonin reuptake inhibitors (SRIs), which are the first-line treatment for OCD, can induce switches, mixed states, or cycle acceleration in BD, especially if administered at high doses and maintained for a long time [11,12]. Available data on the treatment of patients with BD and co-occurring OCD and anxiety disorders show more evidence of benefit with using BD treatments primarily, thus giving hierarchical priority to BD [10,13].

Aripiprazole is a second-generation antipsychotic employed in the treatment of BD: several controlled trials showed its efficacy in all illness phases [14] and its use is recommended by the recent guidelines for the management of patients with BD [15,16]. Moreover, aripiprazole has been shown to be more effective than placebo on clinical symptoms and cognitive functioning in OCD patients who had not responded or not fully responded to SRI treatment alone [17,18,19,20]. Therefore, aripiprazole augmentation to mood stabilizers could represent a promising treatment in BD patients with comorbid OCD, but so far, few data are available. Indeed, only one clinical trial was conducted to study the effects of aripiprazole as an adjuvant treatment for obsessive-compulsive (OC) symptoms in the manic phase of BD, showing good efficacy of aripiprazole in the treatment of OC symptoms [21]. To our knowledge, there are no studies that investigated the efficacy on OC symptoms of aripiprazole augmentation to mood stabilizers in euthymic BD-OCD patients.

The present study aims to assess efficacy on OC symptoms and tolerability of the addition of aripiprazole to a mood stabilizer, either lithium or valproate, in patients with remitted BD and comorbid OCD.

## 2. Materials and Methods

### 2.1. Study Design

The study had a prospective observational design and involved a series of euthymic BD patients with comorbid OCD, treated with lithium or valproate monotherapy, and prescribed aripiprazole for OC symptoms.

Aripiprazole starting dose and dosage changes during the follow-up period were established according to clinical judgment. The mood stabilizer blood levels were monitored monthly and, if necessary, the dosage was adjusted to comply with the blood therapeutic range (valproate: 50–100 mcg/L; lithium: 0.5–0.8 Mmol/L). Each patient was followed-up for 3 months.

Since the observational design of the study, no attempt was made to influence decisions regarding the study treatments and patients received care as usual.

All participants gave written consent before starting treatments. The research was conducted in accordance with the Declaration of Helsinki in its most recent version (64th WMA General Assembly, Fortaleza, Brazil, October 2013). The study was reviewed and approved by the local Ethics Committee (approval code: 7119/Tit:02/Cat:06; approval date: 18 April 2018).

### 2.2. Sample

Study participants were either referred by general practitioners or psychiatrists, or self-referred to the Mental Health Department of Alba and Bra (Cuneo, Italy) from May 2018 to February 2020.

Patients fulfilling the following criteria were included: (a) aged 18 to 70 years; (b) Diagnostic and Statistical Manual of Mental Disorders Fifth Edition (DSM-5) diagnosis of BD type I and II (American Psychiatric Association, 2013); (c) no acute mood episodes in the last 3 months; (d) no current active suicidal ideation or recent (within 6 months) suicide attempts; (e) current lithium or valproate monotherapy in therapeutic range; (f) DSM-5 diagnosis of comorbid OCD, with a Yale–Brown Obsessive-Compulsive (Y-BOCS) Scale total score of 16 or higher; (g) consent to participate in the study.

Exclusion criteria were: (a) a present or previous diagnosis of schizophrenia or other psychotic disorders; (b) concomitant severe, unstable, active neurological or physical diseases; (c) current substance use disorder, except for nicotine; (d) pregnancy or breastfeeding; (e) other pharmacological treatments than lithium or valproate monotherapy; (f) history of nonresponse or intolerance to aripiprazole.

### 2.3. Assessment and Procedures

General socio-demographic and clinical information were collected for each subject by looking at medical reports and by directly interviewing the patients. Patients underwent examinations (control visits) during the follow-up period (3 months) according to clinical practice.

OC symptoms were assessed by means of the Y-BOCS scale at baseline and the end of the follow-up period. All psychiatric diagnoses and clinical assessments were made by certified psychiatrists with at least five years of experience The likelihood of aripiprazole to improve OC symptoms was assessed evaluating the mean change in YBOCS total score from baseline to endpoint. Moreover, qualitative analyses were performed, calculating rates of responders (Y-BOCS reduction of at least 35% from baseline) and partial responders (Y-BOCS reduction greater than 25% but less than 35% from baseline), according to stages of response recently proposed by Mataix-Cols and colleagues [22].

### 2.4. Statistical Analysis

Subjects’ characteristics were summarized as means and SD for continuous variables and as frequencies and percentages for categorical variables.

Given a significance level of 0.05, the post-hoc power analysis on the sample size of 70 subjects showed a statistical power > 90% related to Y-BOCS mean scores.

The normality of data distribution was evaluated using the Kolmogorov-Smirnov test. Since the distribution of Y-BOCS total scores at baseline was not normal (KS: 0.133; *p*: 0.004), the non-parametric Wilcoxon test was used to assess the changes in Y-BOCS total score from baseline to endpoint. For missing values, a “Last Observation Carried Forward” (LOCF) approach was applied.

Further, aripiprazole efficacy and tolerability were compared between two subgroups (patients taking lithium and patients taking valproate), by way of chi-square (χ2) for categorical variables (response rates, percentage of adverse events) and Kruskal–Wallis non-parametric test for continuous variables (mean Y-BOCS scores).

The results from every statistical comparison of the treatment groups were presented as 2-sides *p* values rounded to 3 decimal places. The criterion for statistical significance in all comparisons was a *p*-value < 0.05. All statistical analyses were performed by SPSS software version 26.0.

## 3. Results

Seventy patients fulfilled the study criteria and were enrolled. Thirty-three were males (47.1%) and the mean age of the sample was 42.6 ± 13.6. There was a slight prevalence of patients with BD type II (*n* = 37, 52.9%). Forty-five patients (64.3%) were treated with lithium and 25 (35.7%) with valproate. All baseline demographic and clinical characteristics of the sample are shown in Table 1.

Fifty-five patients completed the 12-week follow-up period. The dropout rate was 21.4% (*n* = 15). Reasons for dropout were consent withdrawal (*n* = 1) and the occurrence of adverse events (AEs) (*N* = 14): AEs that led to dropout are resumed in Table 2. All dropouts were within week 6.

**Figure 1:**
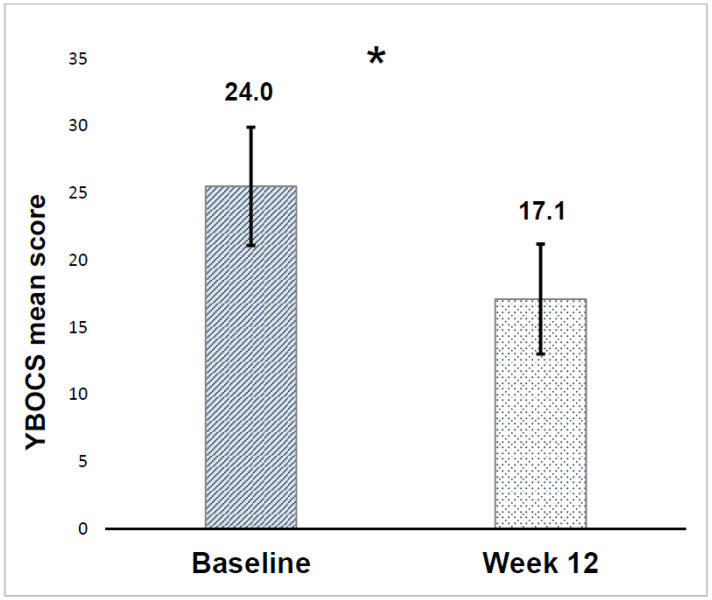
Yale–Brown Obsessive-Compulsive (YBOCS) score mean reduction. *: *p* < 0.001.

Patients who completed the 12-weeks observation period showed an improvement in OC symptoms: Y-BOCS mean score significantly decreased at week 12 (17.1 ± 4.3) compared to baseline (24.0 ± 4.1) (Wilcoxon test: Z: −6.390, *p* < 0.001) (Figure 1).

Likewise, LOCF analysis showed a significant reduction of the Y-BOCS scores from baseline (25.3 ± 4.4) to the endpoint (18.0 ± 4.8) (Wilcoxon test: Z: −6.485, *p* < 0.001). The qualitative analysis highlighted treatment response (Y-BOCS reduction ≥ 35% from baseline) in 23 patients (41.8%) and partial response (Y-BOCS reduction greater than 25% but less than 35% from baseline) in 10 patients (18.2%).

During the follow-up period, no mood recurrence was observed in the sample.

**Figure 2:**
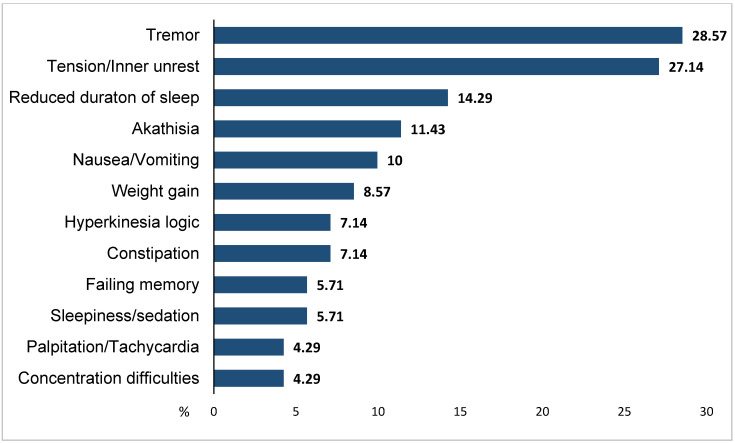
Most common adverse events in completers (*N* = 55). Adverse events < 4%: asthenia/lassitude/increased fatigability, orthostatic dizziness, weight loss, rigidity, orgastic dysfunction, micturition disturbances, erectile dysfunction, ejaculatory dysfunction, paraesthesias, increased tendency to sweating, increased dream activity, diarrhea, increased sexual desire, polyuria/polydipsia.

AEs experienced by subjects who completed the observation period are shown in Figure 2.

Overall, 90.1% of completers (50/55) had at least 1 AE; the most common side effects reported in the total sample were tremor (28.6%), tension/inner unrest (27.1%), reduced duration of sleep (14.3%), and akathisia (11.4%).

The total sample was further analyzed according to whether patients were treated with lithium (*N* = 36) or valproate (*N* = 19). Analyzing completers, lithium and valproate subgroups did not significantly differ in Y-BOCS scores at baseline (23.9 ± 4.2 vs. 24.3 ± 3.9; *p*: 0.804) and week 12 (17.1 ± 4.8 vs. 17.0 ± 3.5; *p*: 0.986). Further, a significant decrease from baseline to week 12 was observed both in the lithium subgroup (Z: −5.166, *p* < 0.001) and in valproate subgroup (Z: −3.793, *p* < 0.001). Concerning the qualitative analysis, we did not find significant differences between the 2 subgroups, neither examining response rates (lithium subgroup: 41.7%; valproate subgroup: 42.1%; *p*: 0.975), nor considering partial response rates (lithium subgroup: 16.7%; valproate subgroup: 21.1%; *p*: 0.134). Results did not change after LOCF analysis. Lastly, with regards to tolerability, patients with at least 1 AE were 40 (89.0%) in the lithium subgroup and 24 (96.0%) in the valproate subgroup (*p*: 0.309).

## 4. Discussion

To our knowledge, this is the first study focusing on the efficacy and tolerability of aripiprazole augmentation to mood stabilizers for the treatment of OC symptoms in euthymic BD patients.

In line with a hierarchical model explaining comorbid psychiatric disorders in BD patients as epiphenomena of the main disease and not as distinct pathologies, we hypothesized that OC symptoms would respond to the addition of a second mood stabilizer. This approach should protect BD patients from the risk of switching into manic or mixed states or of cycle acceleration related to the use of serotonergic agents. Among mood stabilizers, we chose aripiprazole because it’s a first-line agent in the treatment of BD and available data demonstrates its efficacy on clinical symptoms in SRI-resistant OCD patients. Aripiprazole dosage during the follow-up period was established and modified according to clinical judgment. Mean final aripiprazole dosage (15.2 mg/day) was in line with doses used both in studies that demonstrated the efficacy of aripiprazole addition in resistant OCD [17,18,19,20] and in the only study investigating the efficacy of aripiprazole augmentation in BD-OCD patients [21].

Our findings show that aripiprazole addition to lithium or valproate can reduce OC symptoms in real-world BD euthymic patients. Indeed, in our sample at the end of the 12-week observation period, 41.8% of subjects met the criteria for treatment response of OC symptoms and 18.2% showed a partial response, with no difference between patients in lithium or valproate monotherapy. The only study that investigated the efficacy of aripiprazole as an adjuvant treatment for OC symptoms in patients with BD was performed in the manic phase and showed a >34% decrease in mean YBOCS score in more than 90% of subjects treated with aripiprazole (*n* = 21) [21]. However, response rates found in the context of manic or euthymic phases of BD are difficult to compare. Indeed, during an acute manic episode there is a close relationship between OC symptoms and mood elevation; anti-manic agents, including aripiprazole, might be more effective in the treatment of OC symptoms in manic patients rather than in euthymic subjects.

Concerning tolerability, we found high rates of AEs, both in included patients (91.4%) and in completers (90.1%), especially tremor, tension, reduced duration of sleep and akathisia, in line with those most frequently encountered during trials with aripiprazole in patients with BD [23]. Although no severe AEs emerged during the study, one in five patients discontinued the treatment because of the occurrence of side effects. The side effects found in our study are likely to be attributable to the addition of aripiprazole: all adverse events emerged after aripiprazole initiation and there were no differences in tolerability between patients receiving lithium or valproate. However, the impact of drug-drug interactions cannot be excluded. In a multicenter, double-blind, randomized study that evaluated the adjunction of aripiprazole to lithium or valproate as maintenance therapy in BD, the percentage of patients experiencing any AE was lower (75.2%) [24]. The higher prevalence of AEs found in our real-world patient sample could mainly be explained by the fact that strict inclusion and exclusion criteria for randomized controlled trials may exclude the majority of patients encountered in clinical practice [25].

The main limitation of this study is represented by the prospective observational design: our findings require confirmation in double-blind trials. Furthermore, limitations include the relatively small sample size and the absence of a control group. Moreover, we could not run regression models to adjust for potential confounding variables (e.g., baseline YBOSC scores), due to the type of analysis performed and the lack of a control group. Finally, the relatively short length of the observation period (12 weeks) does not allow us to discuss the long-term benefits and risks of this approach; therefore, we aim to continue the follow-up of this cohort for a longer period of time. On the other hand, a point of strength should be taken into account: subjects enrolled for this study were representative of “real-world” patients with BD and comorbid OCD.

## 5. Conclusions

Despite all the limitations, our study is the first to show that a second mood stabilizer, aripiprazole, added to lithium or valproate is effective in reducing OC symptoms in euthymic patients with BD and co-occurring OCD. Given the high prevalence of BD-OCD comorbidity and the negative impact of OCD on clinical course, quality of life, and treatment response of bipolar patients, studies aimed at investigating treatment strategies in this subgroup of difficult-to-treat patients are highly warranted.

## Figures and Tables

**Table 1:** Baseline demographic and clinical characteristics.

Parameters	*N* = 70
**Age**, years (mean ± SD)	42.6 ± 13.6
**Sex**, *n* (%)	
Male	33 (47.1)
Female	37 (52.9)
**Marital status**, *n* (%):	
Never married	33 (47.1)
Married	25 (35.8)
Divorced	11 (15.7)
Widowed	1 (1.4)
**Educational level**, years (mean ± SD)	11.8 ± 3.5
**Currently working**, *n* (%)	
Yes	33 (47.1)
No	37 (52.9)
**Age at onset of bipolar disorder**, years (mean ± SD)	23.8 ± 6.3
**Bipolar disorder subtype**, *n* (%)	
I	33 (47.1)
II	37 (52.9)
**Duration of illness**, years (mean ± SD)	19.1 ± 11.8
**Time from last mood episode**, months (mean ± SD)	21.7 ± 13.4
**Mood stabilizer**, *n* (%)	
Lithium	45 (64.3)
Valproate	25 (35.7)

**Table 2:** Adverse events leading to drop-out.

Patients	Reason for Drop-Out
P1	Hyperkinesia logic; Nausea/vomiting
P2	Akathisia; Reduced duration of sleep; Tremor
P3	Tension/Inner unrest; Akathisia; Reduced duration of sleep; Tremor
P4	Tension/Inner unrest; Akathisia; Nausea/Vomiting; Orthostatic dizziness
P5	Tension/Inner unrest; Nausea/Vomiting
P6	Tension/Inner unrest; Reduced duration of sleep
P7	Tension/Inner unrest; Akathisia; Hyperkinesia logic; Concentration difficulties; Failing memory
P8	Tension/Inner unrest; Reduced duration of sleep
P9	Tension/inner unrest; Akathisia; Reduced duration of sleep; Tremor; Concentration difficulties
P10	Tremor; Rigidity
P11	Tension/Inner unrest; Hyperkinesia logic
P12	Akathisia
P13	Tension/Inner unrest; Akathisia; Reduced duration of sleep; Tremor; Hyperkinesia logic;
P15	Tension/Inner unrest; Akathisia; Reduced duration of sleep; Nausea/Vomiting

Mean ± SD final aripiprazole dose was 15.2 ± 5.3 in the completer sample (*N* = 55).

## Data Availability

The data that support the findings of this study are not openly available, due to confidentiality of data collected from hospital clinical charts.
